# Novel peroral cholangioscopy-directed lithotripsy using an ultraslim upper endoscope for refractory Mirizzi syndrome: A case report

**DOI:** 10.1097/MD.0000000000022649

**Published:** 2020-11-06

**Authors:** Jian Li, Shao-Ju Guo, Jing-Chao Zhang, Hong-Yan Wang, Kai Li, Cheng-Shan Xu, Xue-Fang Wang

**Affiliations:** Endoscopy Unit, Department of Gastroenterology, Shenzhen Traditional Chinese Medicine Hospital, The Fourth Clinical Medical College of Guangzhou University of Chinese Medicine, Shenzhen, China.

**Keywords:** direct peroral cholangioscopy, gallstone impaction, hybrid anchoring balloon, Mirizzi syndrome, ultraslim endoscope

## Abstract

**Rationale:**

Mirizzi syndrome (MS) is an uncommon condition characterized by common hepatic duct (CHD) compression by an impacted gallbladder or cystic duct stones or adjacent inflammation. To date, a standardized therapeutic strategy for MS has not been established yet, owing to its complex clinical presentation. Thus, researchers still have to develop new optimized approaches to solve this problem. Herein, we describe a patient with refractory MS who underwent a successful treatment by novel hybrid anchoring balloon-guided direct peroral cholangioscopy (POC) using an ultraslim endoscope.

**Patient concerns:**

A 56-year-old man with a history of biliary stone was referred to our hospital for complaints of discomfort in the right upper quadrant of the abdomen and obstructive jaundice. Endoscopic retrograde cholangiopancreatography showed an 18-mm impacted stone at the level of the cystic duct, which compressed the CHD. The CHD had local stricture, with its upstream and intrahepatic bile duct dilation.

**Diagnoses:**

He was diagnosed with type I MS.

**Interventions:**

Initially, the patient received an endoscopic major sphincterotomy. However, conventional stone extraction, including mechanical lithotripsy, was unsuccessful. Then, after signing the informed consent form for further treatment, he was successfully treated with novel hybrid anchoring balloon-guided direct POC.

**Outcomes:**

The patient had no operative complications and was discharged with cleared ducts. At the 3-year follow-up, he was asymptomatic.

**Lessons:**

Our novel hybrid anchoring balloon-guided direct POC may be an effective alternative treatment approach for difficult gallbladder cases, such as refractory MS.

## 1 Introduction

Mirizzi syndrome (MS) is a challenging complication of chronic cholelithiasis for the biliary surgeon, and impacted stones in the cystic duct or gallbladder lead to common hepatic duct (CHD) obstruction by either extrinsic compression or inflammation.^[1]^ Minimally invasive techniques, such as laparoscopic cholecystectomy and endoscopic retrograde cholangiopancreatography (ERCP), are generally the preferred treatment.^[2]^ However, performing these procedures may not always be clinically possible because of the distorted anatomy and dense adhesions from chronic inflammation that are commonly encountered at the Calot triangle. Direct peroral cholangioscopy (POC) using an ultraslim endoscope has been proposed as a potential solution for various biliary diseases, because it allows direct endoscopic visualization of the bile duct.^[3]^ We previously observed that the use of a novel hybrid anchoring balloon device with an ultraslim endoscope performed well in facilitating direct POC for extracting large impacted common bile duct (CBD) stones.^[4]^ Here, we describe a patient with refractory MS who underwent successful treatment by novel hybrid anchoring balloon-guided direct POC using an ultraslim endoscope.

## 2 Case report

This report was reviewed and approved by the ethics committee of the Shenzhen Traditional Chinese Medicine Hospital, Shenzhen, China. Written informed consent was obtained from the patient to publish his case and all accompanying images.

**Figure 1:**
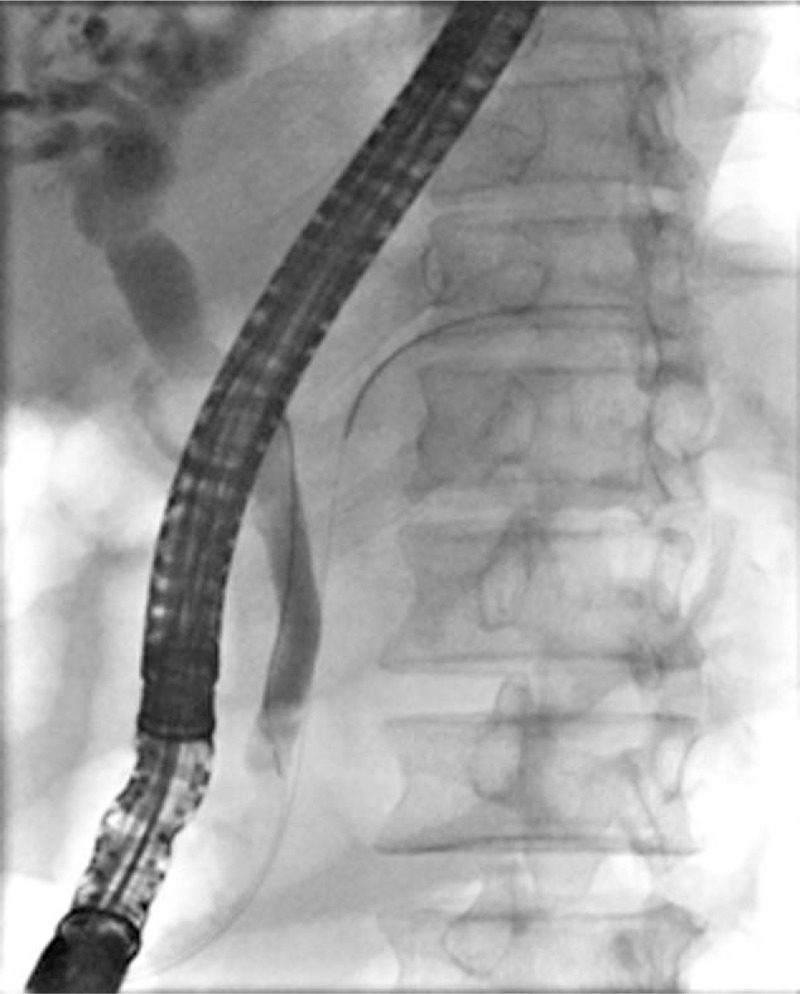
Radiograph showing an 18-mm gallstone compressing onto the CHD at the level of the cystic duct, with its upstream CHD and intrahepatic bile ducts dilation. CHD = common hepatic duct.

A 56-year-old man presented to our hospital with discomfort in the right upper quadrant of the abdomen and obstructive jaundice. Abdominal computed tomography performed at a local clinic showed a biliary stone in the CBD. Physical examination on admission revealed unremarkable findings, apart from the moderate yellowing of the sclera and tenderness in the upper abdomen. Laboratory test results showed elevated levels of the white blood cell count and C-reactive protein. Moreover, the liver function test revealed the following abnormal results: total bilirubin, 157.8 μmol/L; direct reacting bilirubin, 99.7 μmol/L; aspartate aminotransferase (AST), 185.1 IU/L; alanine aminotransferase (ALT), 223.7 IU/L; and γ-glutamyltransferase. 361.0 IU/L. Then, ERCP showed an 18-mm impacted stone at the level of the cystic duct, which compressed the CHD. The CHD had local stricture with its upstream and intrahepatic bile duct dilation (Fig. 1). There were no abnormal changes observed in the CBD and duodenal papilla. Based on these findings, we highly suspected that the patient had type I MS; thus, endoscopic major sphincterotomy was scheduled. Conventional stone extraction, including mechanical lithotripsy, was unsuccessful owing to the inability to access the location above the impacted stone with an extractor balloon or a basket. Therefore, after the patient provided informed consent for direct POC, we further performed an endoscopic treatment.

**Figure 2:**
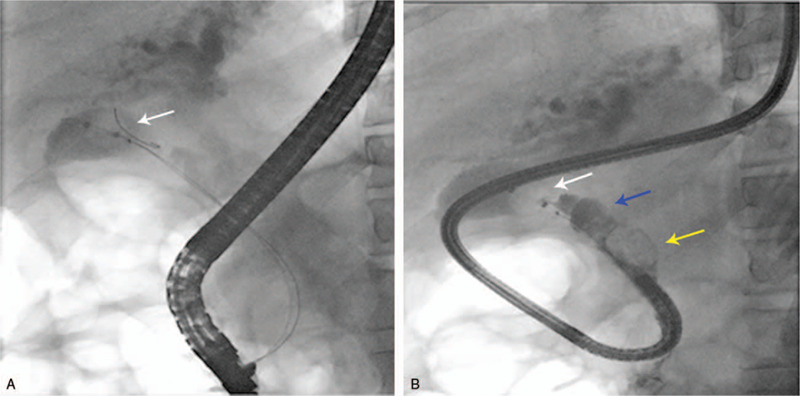
A, Radiograph showing a hybrid anchoring balloon device (white arrow) anchored in the gallbladder through the duodenoscope. B, Radiograph showing an ultraslim upper endoscope that has been passed over the anchored hybrid balloon device and advanced into the cystic duct. The impacted stone (yellow arrow), the dilated cystic duct (blue arrow), and the anchored balloon (white arrow) of the device are seen in these pictures.

**Figure 3:**
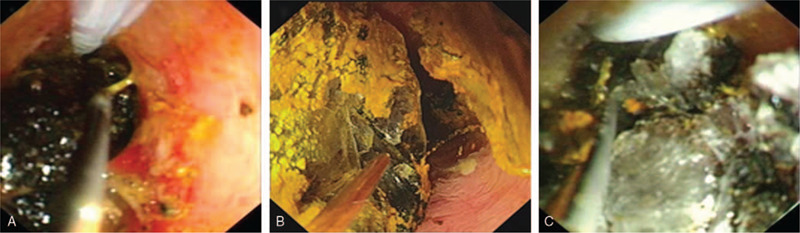
A, Cholangioscopic image showing an ultraslim upper endoscope advancing into the cystic duct by the hybrid anchoring balloon device. B and C, Cholangioscopic image showing EHL under direct POC guidance. The EHL probe (yellow), the catheter (white), and the guidewire (black) of the hybrid anchoring balloon device are seen in these pictures. EHL = electrohydraulic lithotripsy, POC = peroral cholangioscopy.

**Figure 4:**
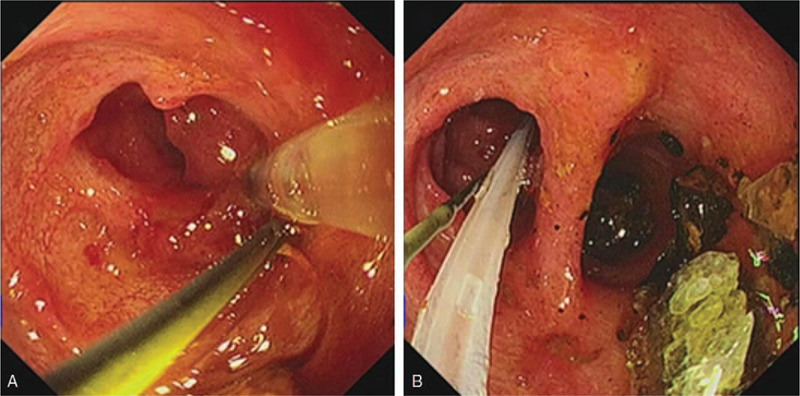
A, Cholangioscopic image showing the spiral valve structure of the cystic duct with no stone remnants. B, Cholangioscopic image showing clearance of the stones at the cystic duct level (left) and fragmented stones in CBD to be extracted with a basket and balloon. CBD = common bile duct.

The direct POC technique was performed as previously described.^[4]^ In brief, after performing balloon dilatation of the papilla (to 12 mm in diameter), a 0.035-inch guidewire (Loop tip; Cook Medical Co, Winston-Salem, NC) was placed within the gallbladder to maintain access. The hybrid anchoring balloon-guided device, with a 0.021-inch guidewire tied to the balloon catheter at 2 to 3 mm from its distal end, was inserted into the working channel of the duodenoscope and inflated to anchor it within the gallbladder (Fig. 2A). Thereafter, the 0.035-inch guidewire was withdrawn from the gallbladder, and the most proximal handle was detached from the catheter of the device. Next, the duodenoscope was removed, leaving the anchored hybrid balloon-guided device behind. Subsequently, an ultraslim endoscope (GIF-XP290N, 5.4-mm outer diameter; Olympus, Tokyo, Japan) was advanced into the cystic duct over the 0.021-inch guidewire of the device (Figs. 2B and 3A). An electrohydraulic lithotripsy (EHL) probe (Lithotrips-Y27080B; Karl Storz, Tuttlingen, Germany) was passed through the ultraslim endoscope to break the stone (Fig. 3B and C). Finally, stone clearance was achieved at the cystic duct and CBD with a basket and balloon (Fig. 4A and B).

The total operative time of this novel POC-directed lithotripsy was 80 minutes. No operative complications, such as bleeding, cholangitis, pancreatitis, aspiration, pneumonia, and perforation, occurred. The patient's jaundice faded postoperatively, and he was discharged with cleared ducts. There was no recurrence at the 3-year follow-up assessment.

## 3 Discussion

MS is a rare complication of chronic cholecystitis, and its minimally invasive treatment, including laparoscopic cholecystectomy and ERCP, is usually associated with patient benefits, such as pain reduction and shortened length of recovery.^[2]^ Laparoscopic cholecystectomy is a viable treatment choice, as endoscopic treatment is safe and efficient.^[5]^ However, in difficult gallbladder cases, the laparoscopic approach is challenging because of the presence of impacted stones at the level of the cystic duct, adjacent to the CHD and CBD. Additionally, these cases are usually accompanied by local inflammation and dense adhesion, increasing the risk of accidental bile duct injury during dissociation, dissection, and suturing of the incision, which cause failure of the laparoscopic treatment. Besides, conventional endoscopic techniques in ERCP can provide an alternative means of treatment and stone removal.^[6]^ Notwithstanding this, such techniques often fail to extract the impacted stones found in MS. Since the cause of MS is the impacted gallstones in the cystic duct or gallbladder neck, neither a basket nor a balloon can easily obtain access to the location above a stricture or can achieve a maximum stretch to cover the stone.

Other means of stone fragmentation in MS have to be sought. Recently, a study indicated that choledochotomy and intraoperative cholangioscopy with EHL can be successfully performed in cases with difficult gallbladder access, such as MS.^[7]^ Studies have also examined the clinical feasibility of fragmentation techniques in direct POC under endoscopic visualization of the bile duct. The dedicated “mother–baby” endoscopic system has expedited the processing of biliary insertion, but it also has some disadvantages, including a relatively small working channel, low-quality endoscopic imaging, and high cost.^[8]^ The SpyGlass DS Direct Visualization System might be a potential solution for some of these deficiencies, although its use is limited only to specialized centers because of its high cost. Thus, to solve these problems, the direct POC method with a single-operator platform using an ultraslim endoscope provides another alternative treatment option in MS cases. It provides high-quality endoscopic imaging, a large working channel with 2-mm diameter, and comparatively low cost, even though its biliary insertion rates are relatively low and inconsistent.^[9]^ However, to date, there have been limited reports on the use of the direct POC method as a simple and easy treatment for MS, as described in this study.^[2,10]^

Our report illustrates a patient with refractory MS who was successfully treated with endoscopic techniques in direct POC using an ultraslim endoscope, with the assistance of a hybrid anchoring balloon to facilitate stable access and maintain the desired endoscope position for subsequent procedures. In general, our specialized hybrid anchoring balloon accessory, which was derived from the intraductal balloon-guided method, has greatly enhanced the performance of direct POC.^[4,11]^ In this case, the balloon of the device used was solidly anchored within the gallbladder so that the lumen was large enough for the balloon to inflate to 20 mm in diameter without causing injury to the gallbladder wall. Additionally, our hybrid anchoring balloon device performed well in maintaining good stability and maneuverability of the ultraslim endoscope during the whole procedure. Given that our device provides a 2.0-mm working channel for EHL probe insertion, irrigation, and 0.021-inch guidewire insertion, there is no need to withdraw the ultraslim endoscope repeatedly for a subsequent procedure once it is anchored within the gallbladder.

Of note, we suggest using this direct POC method only in patients with both cystic duct and CBD diameters of ≥5 mm, provided that the biliary passage is large enough to allow the insertion of an ultraslim endoscope to access the desired position. Although no severe complications were observed in our case, our novel technique has several limitations. Compared to the SpyGlass technique, our method requires endoscopists to undergo a relative more complex procedure, such as advancing a duodenoscope first and then exchanging with an ultraslim endoscope to achieve a technical access. On the other hand, since bile duct injury always occurs during the EHL process, we recommend that experienced and skilled operators should perform the direct POC procedure, especially in complex interventional cases.

In conclusion, our novel hybrid anchoring balloon-guided direct POC may be an effective and feasible treatment approach for difficult gallbladder cases, such as refractory MS. However, because of the lack of standardized care regarding the endoscopic treatment of MS, the choice of endoscopic technique is subject to careful assessment of the patients’ anatomy and medical conditions.

## Acknowledgments

The authors thank Editage colleague (Shanghai, China) for their language edits.

## Author contributions

**Conceptualization:** Jian Li, Hong-Yan Wang.

**Data curation:** Kai Li, Cheng-Shan Xu, Xue-Fang Wang.

**Investigation:** Hong-Yan Wang, Jing-Chao Zhang.

**Methodology:** Jian Li, Shao-Ju Guo, Jing-Chao Zhang.

**Project administration:** Hong-Yan Wang.

**Supervision:** Shao-Ju Guo.

**Writing – original draft:** Jian Li, Hong-Yan Wang.

**Writing – review &editing:** Jian Li, Hong-Yan Wang.
